# Homocysteine level, body mass index and clinical correlates in Chinese Han patients with schizophrenia

**DOI:** 10.1038/s41598-020-72934-3

**Published:** 2020-09-30

**Authors:** Yuanyuan Huang, Kai Wu, Hehua Li, Jing Zhou, Dongsheng Xiong, Xia Huang, Jiahui Li, Ya Liu, Zhilin Pan, David T. Mitchell, Fengchun Wu, Xiang Yang Zhang

**Affiliations:** 1 Department of Psychiatry, The Affiliated Brain Hospital of Guangzhou Medical University (Guangzhou Huiai Hospital), 36 Mingxin Rd, Liwan District, Guangzhou, 510370 China; 2 Department of Biomedical Engineering, School of Materials Science and Engineering, South China University of Technology(SCUT), Guangzhou, China; 3 Guangdong Engineering Technology Research Center for Translational Medicine of Mental Disorders, Guangzhou, China; 4 Guangdong Engineering Technology Research Center for Diagnosis and Rehabilitation of Dementia, Guangzhou, China; 5 National Engineering Research Center for Tissue Restoration and Reconstruction, South China University of Technology, Guangzhou, China; 6 Key Laboratory of Biomedical Engineering of Guangdong Province, South China University of Technology, Guangzhou, China; 7 Department of Nuclear Medicine and Radiology, Institute of Development, Aging and Cancer, Tohoku University, Sendai, Japan; 8 Department of Psychiatry and Behavioral Sciences, The University of Texas Health Science Center At Houston, Houston, TX USA; 9 CAS Key Laboratory of Mental Health, Institute of Psychology, Chinese Academy of Sciences, 16 Lincui Rd, Chaoyang District, Beijing, 100101 China

**Keywords:** Biochemistry, Biomarkers, Diseases

## Abstract

Obesity is common comorbidity in patients with schizophrenia. Previous studies have reported that homocysteine (Hcy) is increased in schizophrenia. However, no study has reported the association between BMI and Hcy levels in schizophrenia. This cross-sectional naturalistic study aimed to evaluate the relationship between BMI, Hcy and clinical symptoms in Chinese Han patients with chronic schizophrenia. Clinical and anthropometric data as well as plasma Hcy level and glycolipid parameters were collected. Psychopathology was measured with the Positive and Negative Syndrome Scale (PANSS). Our results showed that compared with the low BMI group, the high BMI group had a higher PANSS general psychopathology subscore, higher levels of blood glucose, total cholesterol and high-density lipoprotein (HDL) cholesterol (all *p* < 0.05). Hcy levels were negatively associated with BMI in patients (*p* < 0.001). Hcy level, the PANSS general psychopathology subscale, total cholesterol and education (all *p* < 0.05) were the influencing factors of high BMI. Our study suggest that Hcy level may be associated with BMI in patients with schizophrenia. Moreover, patients with high BMI show more severe clinical symptoms and higher glucose and lipid levels.

## Introduction

Schizophrenia is a severe psychiatric disorder characterized by chronic progressive impairments of physical, cognitive and psychosocial function. Weight gain is common in schizophrenia, which is a risk factor for some chronic diseases, such as diabetes, hypertension, and cardiovascular disease^1–4^. In addition, obesity leads to a lower quality of life and 10 ~ 20 years of shorter life expectancy in schizophrenia^5^. Most studies have shown that weight gain in psychiatric patients is associated with lifestyle^6,7^, metabolic abnormalities^8,9^, and use of antipsychotic medications^10^.

Homocysteine (Hcy), a sulfur-containing amino acid, participates in the methionine cycle that influences brain development through multiple cellular pathways^11^. There is growing evidence that Hcy levels are closely related to neuropsychiatric disorders such as Alzheimer disease^12^, affective disorders, and schizophrenia^13–16^. Numerous studies reported that Hcy levels were increased in patients with acute and chronic schizophrenia^17,18^. A previous study found that a 5-μmol/l increase in Hcy levels increased the risk of schizophrenia by up to 70%^19^. Further research found that Hcy levels were positively correlated with the severity of negative symptoms^18^, duration of untreated psychosis^20^, and cognitive deficits^21^ in patients with schizophrenia.

Evidence for the association between different BMI and Hcy levels in the general population have been presented, with a particular focus on the course of physical disease^22,23^. However, results on the relationship between BMI and Hcy levels showed conflicting data^24^. A recent meta-analysis reported that Hcy was positively correlated with obesity^25^, while a recent study demonstrated opposite results in the general population^26^. A recent cross-sectional study showed that Chinese bipolar disorder (BD) patients with hyperhomocystinemia had elevated BMI^16^. Taken together, it appears that Hcy may play a role in high BMI or obesity in both healthy population and patients with psychiatric disorders.

Based on the higher prevalence of obesity and increased Hcy levels in patients with schizophrenia and the close relationship between Hcy and obesity, it is of great interest to explore their possible association in schizophrenia. To our best knowledge, no study has reported the association between BMI and Hcy levels in patients with schizophrenia. Therefore, the main purposes of this study were to bridge this gap by examining: (1) whether there were differences in plasma Hcy, clinical data, glucose and lipid metabolism parameters between high and low BMI groups in patients with schizophrenia; (2) whether there were a correlation between BMI, Hcy and clinical symptoms in Chinese Han patients with schizophrenia.

## Methods

### Subjects

Using a cross-sectional naturalistic design, 120 patients with schizophrenia were recruited from the Affiliated Brain Hospital of Guangzhou Medical University, a psychiatric teaching hospital with more than 120 years of history in Guangzhou city. The study was conducted from June, 2018 to May, 2019. Inclusion criteria included the following: (1) aged 18–60 years, Han Chinese; (2) confirmed DSM-V diagnostic criteria of schizophrenia by two experienced psychiatrists according to the Structured Clinical Interviewed for DSM-V (SCID); (3) a stable dose of antipsychotic drugs for ≥ 4 weeks before the start of the study.

Each subject underwent a complete medical history, physical examination, and laboratory tests. Any subjects with major medical abnormalities were excluded, including acute or unstable medical illnesses or any organic brain diseases. Also, subjects suffering from drug or alcohol abuse/dependence were excluded. The trained research staff collected general information and social-demographic characteristics through questionnaires. Other information, including a history of mental illness and medications were collected from medical records.

### Clinical measurements

The Positive and Negative Syndrome Scale (PANSS) was used to evaluate the psychiatric symptoms of all patients, which was assessed by two psychiatrists who had received special training to ensure consistency. The inter-rater correlation coefficient (ICC) of PANSS total score remained above 0.8 through repeated evaluation.

### BMI measurements

BMI [weight (kg) / height squared (m^2^)] was calculated in a standardized fashion by assessing body weight and height. Height was measured when the subject was barefoot and upright. Weight was measured by a 0.1 kg electronic scale when the subjects wore light indoor clothing. Referring to the Chinese guidelines for adults^27^, it was considered as overweight with 24 ≤ BMI < 28 kg/m^2^, and BMI ≥ 28 kg/m^2^ was classified as obese. In this study, all patients were divided into high BMI group (BMI ≥ 24 kg/m^2^) and low BMI group (BMI < 24 kg/m^2^).

### Glycolipid metabolism and Hcy analysis

Venous blood was collected in the early morning from each patient after at least 8 h of fasting. The blood was collected with an EDTA tube and centrifuged (3000r/m) for 10 min within 30 min of collection. The plasma was isolated and stored at −80 °C for testing.

The levels of plasma glucose, hemoglobin A1c (HbA1c), triglycerides (TGs), total cholesterol (TC), high-density lipoprotein (HDL) cholesterol, and low-density lipoprotein (LDL) cholesterol were measured by commercially available kits from Beijing Leadman Biotechnology Co. Ltd. (Beijing, China) and by Beckman AU480 automatic biochemical analyzers (Olympus, Japan).

The Hcy levels were measured by Hcy assay kits (enzyme cycle assay), which were purchased from Beijing Leadman Biochemistry Co.Ltd. The assay method was in strict accordance with the instructions of the kit.

All biochemical analyses were performed by a technician in the clinical laboratory center of the hospital, who was blind to the status of subjects.

### Statistical analysis

Demographic and clinical characteristics between groups were compared using analysis of variance (ANOVA) for continuous variables and chi-squared tests for categorical variables. Since TG and Hcy levels did not follow a normal distribution (Kolmogorov–Smirnov one-sample test, *p* < 0.05), we first log transformed the data to a normal distribution and then used the transformed values in subsequent statistical tests. Further analysis of covariance (ANCOVA) was performed to control for the confounding factors including education, sex, age, age of onset and antipsychotic treatment (type, dose and duration of treatment). Associations between variables were examined by using Pearson correlation coefficients. We adopted Bonferroni correction to control for multiple tests. Multivariate regression analysis (enter model) was used to assess significant factors associated with BMI after controlling for variables. SPSS version 18.0 was used for all statistical analyses. All *p* values were defined with a 2-tailed significance level at 0.05.

## Results

### Demographics characteristics

A total of 120 patients including 76 males and 44 females were recruited. Their average age was 46.92 ± 4.41 years and the mean education level was 11.05 ± 3.59 years. The average illness duration was 256.85 ± 155.42 months and the age of onset was 25.94 ± 9.16 years.

Socio-demographic data and clinical profiles of patients with high BMI (n = 51) and low BMI (n = 69) groups are displayed in Table 1. There was no significant difference in demographic and clinical characteristics between the two groups (all *p* > 0.05), except for the education level and BMI (both *p* < 0.001; Bonferroni corrected both *p* < 0.01).

**Table 1:** Socio-demographic data and clinical profiles of schizophrenia patients with high or low BMI.

	All subjects	Subjects with high BMI	Subjects without low BMI	*F/x*^2^	*p* value
	N = 120	N = 51	N = 69		
Age, mean (SD), years	46.92 (4.41)	47.88 (12.55)	46.20 (13.15)	0.50	0.482
Education, mean (SD), years	11.05 (3.59)	12.49 (3.45)	9.99 (3.32)	16.13	< 0.001**
Gender, male/female, n / n	76 / 44	32 / 19	44 / 25	0.01	0.908
Family history, n (%)	29 (24.37%)	14 (27.45%)	15 (21.74%)	0.52	0.521
Married, n (%)	76 (63.86%)	20 (39.22%)	24 (34.78%)	0.25	0.702
Smoking, n (%)	22 (18.49%)	10 (19.61%)	12 (17.39%)	0.10	0.814
BMI, mean (SD), kg/m^2^	24.27 (4.41)	28.24 (2.82)	21.33 (2.76)	179.87	< 0.001**
Age of onset, mean (SD), years	25.94 (9.16)	26.58 (10.02)	25.46 (7.79)	0.42	0.519
Duration of illness, mean (SD), days	256.85 (155.42)	274.45 (151.17)	243.58 (158.41)	1.10	0.296
Clozapine / Olanzapine, n (%)	41 / 27 (56.67%)	17 / 9 (50.98%)	24 / 18 (60.87%)	1.20	0.377
**PANSS scores**					
PANSS Total, mean (SD)	58.59 (16.71)	59.94 (18.95)	57.59 (14.90)	0.58	0.449
P subscore, mean (SD)	11.72 (5.45)	11.35 (5.67)	12.00 (5.33)	0.41	0.523
N subscore, mean (SD)	17.72 (8.13)	17.73 (8.32)	17.71 (8.05)	0.00	0.992
G subscore, mean (SD)	29.19 (7.78)	30.86 (8.84)	27.96 (6.71)	4.19	0.043*

*BMI* body mass index, *PANSS* positive and negative symptoms scale, *P* positive symptom, *N* negative symptom, *G* general psychopathology syndrome.

### Clinical symptoms in high versus low BMI groups

The high BMI patients had higher PANSS general psychopathology subscore than the low BMI patients (*p* < 0.05; Bonferroni corrected *p* > 0.05). When education, sex, and age were added as covariates, there was still a significant difference in PANSS general psychopathology subscore (F_1,118_ = 4.013, *p* = 0.049, r^2^ = 0.008). There was no significant difference in PANSS total, positive symptom or negative symptom scores between the two groups (all *p* > 0.05).

In addition, since the number of patients treated with clozapine/olanzapine was imbalanced between the two groups (50.98%% vs. 60.87%), we performed ANCOVA with antipsychotic type as a covariate. The result still showed significant difference in PANSS general psychopathology subscore (F_1,118_ = 4.331, *p* = 0.040, r^2^ = 0.012) (Table 1).

### Homocysteine and glycolipid metabolism in high versus low BMI groups

As shown in Table 2, compared to the low BMI group, the high BMI group had significantly higher levels of glucose (F_1,118_ = 5.812, *p* = 0.017), total cholesterol (F_1,118_ = 4.921, *p* = 0.028), and HDL cholesterol (F_1,118_ = 4.647, *p* = 0.033), but lower Hcy level (F_1,117_ = 26.011, *p* < 0.001). After controlling for covariates including antipsychotic drugs, there were significant differences in HDL cholesterol (F_1,118_ = 4.174, *p* = 0.044, r^2^ = 0.073) and Hcy levels (F_1,118_ = 23.721, *p* < 0.001, r^2^ = 0.183) between the high and low BMI groups. However, no significant differences in glucose, HbA1c, TGs, and LDL cholesterol were found between two groups (all *p* > 0.05).

**Table 2:** Homocysteine level and cardo-metabolic profiles of schizophrenia patients with high or low BMI.

	Subjects with high BMI	Subjects without low BMI	*F*	*df*	*p* value
	N = 51, mean (SD)	N = 69, mean (SD)			
**Cardo-metabolic**					
Glucose, mmol/L	5.23 (1.17)	4.70 (1.21)	5.812	1,118	0.017*
HbA1c, %	5.45 (0.52)	5.55 (0.86)	0.278	1118	0.599
TG, mmol/L	1.65 (1.09)	1.64 (0.80)	0.002	1,118	0.960
TC, mmol/L	4.76 (1.09)	4.36 (0.86)	4.921	1,118	0.028*
HDL, mmol/L	1.42 (0.20)	1.23 (0.52)	4.647	1,118	0.033*
LDL, mmol/L	2.91 (0.93)	2.65 (0.73)	2.981	1,118	0.087
Homocysteine, mmol/L	14.04 (8.17)	22.19 (14.43)	26.011	1,117	< 0.001**

*TG* Triglyceride, *TC* Total cholesterol, *HDL* high density lipoprotein, *LDL* low density lipoprotein, *HbA1c* Hemoglobin A1c. Triglyceride and homocysteine: data were compared after logarithmic transformation, and descriptive data were original data.

### Relationship between BMI, Hcy, clinical symptoms and glycolipid metabolism

**Figure 1:**
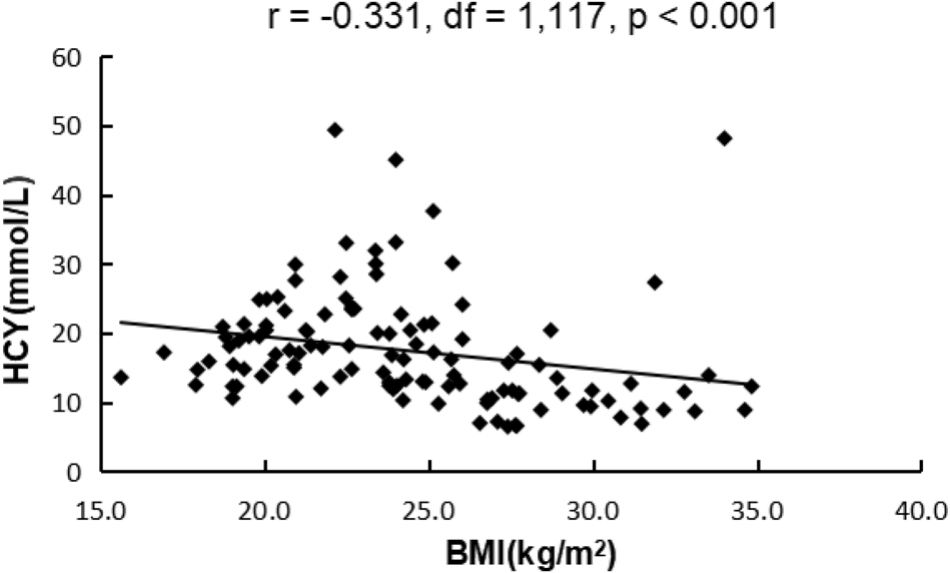
Correlation analysis showed that HCY levels were negatively associated with BMI.

Pearson correlation analyses showed that BMI was negatively associated with Hcy (r = -0.331, df = 1,117, *p* < 0.001), but positively with glucose (r = 0.263, df = 1,118, *p* = 0.004), total cholesterol (r = 0.228, df = 1,118, *p* = 0.001) or HDL cholesterol (r = -0.180, df = 1,118, *p* = 0.05). However, only the significant association between BMI and Hcy passed the Bonferroni correction (*p* < 0.01) (Fig. 1). In addition, glucose level was positively correlated with positive subscore (r = 0.223, *p* = 0.015; Bonferroni corrected *p* > 0.05) and HDL (r = 0.194, *p* = 0.034; Bonferroni corrected *p* > 0.05).

Multiple regression analysis showed that Hcy levels (β = -3.789, t = -2.542, *p* = 0.012), the PANSS general psychopathology subscale (β = 0.103, t = 2.092, *p* = 0.039), total cholesterol (β = 0.979, t = 2.518, *p* = 0.013) and education (β = 0.293, t = 2.817, *p* = 0.006) were independent contributors to the BMI after controlling for age, gender, glucose, and HDL cholesterol and antipsychotic drugs (Table 3).

**Table 3:** Predictors generated by multiple linear regression with BMI as response variable.

	Coefficients		t	*p* value	95.0% confidence interval for B	
	B	S.E			Lower bound	Upper bound
Homocysteine	− 3.789	1.491	− 2.542	0.012	− 6.743	− 0.836
G subscore	0.103	0.049	2.092	0.039	0.005	0.201
Total cholesterol	0.979	0.389	2.518	0.013	0.209	1.749
Education	0.293	0.104	2.817	0.006	0.087	0.5

## Discussion

To our best knowledge, this is the first study to examine the association between Hcy levels and BMI in patients with schizophrenia. The main findings of this study included: (1) The level of Hcy was significantly lower in the high BMI group than that in the low BMI group in patients with schizophrenia. (2) The high BMI patients had higher glucose and lipid levels than low BMI patients. (3) There were significant associations between BMI, Hcy, clinical symptoms and glycolipid metabolism in patients with chronic schizophrenia.

Our study showed that Hcy levels were significantly lower in the schizophrenia patients with high BMI, and that Hcy levels were negatively associated with BMI, which were consistent with some previous studies reporting a negative correlation between Hcy and BMI in the general population^28,29^. Also, a recent large sample of study including 5300 overweight/obese individuals and 5807 normal weight individuals showed that Hcy levels were significantly lower in overweight/obese persons than normal weight controls^26^. However, some previous studies have reported inconsistent results about the relationship between Hcy levels and obesity. For example, some studies showed that compared to non-obese subjects, the Hcy levels were significantly greater in obese subjects suffering from physical disease, such as coronary artery disease and polycystic ovaries^30–32^. The inconsistent results may be due to the differences in subjects that were from different diseases, regions, races, or genetic backgrounds. Other demographic and clinical factors such as diet habits, physical activity, drug treatment, chronic inflammation or the methods of measuring Hcy may also be implicated in the these inconsistent results. Interestingly, we found that Hcy was negatively associated with BMI in schizophrenia, which was inconsistent with most of previous studies showing that high Hcy may be related to obesity and increase the risk of coronary heart disease^33,34^. This may be attributed to the effects of different diseases, different stages of disease development^18^, or medication treatment regiments^35^. Petronijević et al.^18^ proposed that schizophrenia patients had increased plasma Hcy levels in the acute phase, but Hcy levels were significantly decreased after one month of antipsychotic treatment in the remission phase. Several drugs such as antiepileptic drugs^36^, fibrates^37^ and antipsychotic drugs^35^ may interfere with the one-carbon metabolic pathway, leading to changes in Hcy levels^36^. We speculated that the changes in Hcy levels observed in this study may be due to antipsychotic treatment and altered symptoms in patients with schizophrenia. Moreover, some researchers found that an increase in Hcy concentration was associated with an increase in the percentage of adipose tissue^38^, which regulated body fat through epigenetic inheritance of gene expression, leading to the occurrence and development of obesity^31,39^. Conversely, a previous study reported that Hcy had a negative association with both fat mass and lean mass during weight loss^40^. BMI, as a proxy for general obesity, cannot distinguish lean mass and adipose tissue, which may also confuse the relationship between obesity and HCY. Although the mechanism was still unclear, these results suggest that the Hcy metabolic pathway may play an important role in obesity^25^. In addition, we found that Hcy level was negatively correlated with blood glucose and HDL, which is consistent with previous reports^41^ that hyperhomocysteinemia (HHcy) was associated with low levels of HDL. Also, Hcy was associated with glucose metabolism, lipid metabolism, hyperglycemia^42^, and hyperlipidemia^43^.

Another important finding of our study was that obese patients had more severe clinical symptoms, showing that the patients with high BMI had higher PANSS general psychopathology subscore than those with low BMI, and BMI was positively associated with PANSS general psychopathology subscore, which is in line with the finding of previous studies^2,44^. Patients with schizophrenia who had greater severity of psychopathological symptoms may directly affect their enthusiasm for activity, leading to more significant obesity^45^. However, some previous studies have reported opposite results, showing that weight gain was associated with less psychopathological symptoms^46,47^, or that there was no correlation between PANSS and BMI in patients with schizophrenia treated with multiple antipsychotic drugs for one year^44^. Kemp et al. also found that some complex factors, such as medical adherence and treatment time may confuse the results^48^. In addition, several studies also showed that weight gain may be due to treatment of antipsychotic medications, including clozapine, olanzapine, and haloperidol^48–50^. In order to rule out the effects of certain drugs, we included the antipsychotic treatment as covariates and found that there was still a significant correlation, suggesting that the relationship between high BMI and clinical psychopathological symptoms may be more than just a matter of antipsychotic treatment. Although the results were inconsistent in these studies, they all indicated that high BMI showed a certain correlation with clinical symptoms; however, more in-depth studies are needed to explore their causal relationship.

Several related factors for obesity were also identified in patients with schizophrenia in our study. We found that BMI was associated with glycolipid metabolism, showing that compared with low BMI patients, high BMI patients had higher blood glucose and lipid levels, especially total cholesterol, which is consistent with most of previous studies^51–53^. Moreover, a recent study also reported that BMI was positively associated with TGs in Chinese patients with schizophrenia^54^. Also, we found that high BMI was inversely correlated to education levels, which is consistent with previous studies^2,55,56^. In addition, most of the previous studies demonstrated that atypical antipsychotic treatment may lead to increased obesity in schizophrenia^57,58^. In this study, however, we did not find that BMI was significantly associated with clozapine/olanzapine treatment. Clinically, psychiatrists were cautious in the use clozapine or olanzapine to high BMI patients, often switching to other antipsychotic drugs when a patient was becoming obese. Perhaps this is the main reason that we did not find significant association between obesity and clozapine/olanzapine treatment.

There are several methodological limitations of this study. First, a cross-sectional study design cannot directly show the causal relationship between obesity and Hcy in schizophrenia patients. Second, most of the subjects in this study were inpatients and some of them had been in the hospital for a long time. In general, they had more severe negative symptoms, a longer courses of disease, and a long history of antipsychotic treatment, which may have influenced their Hcy levels^35^. Therefore, our findings in this study should be confirmed in a large sample of patients with first-episode and drug-naïve schizophrenia. Third, there are some other confounding factors that may affect Hcy levels and obesity, such as physical exercise, diet, nutrition and psychotropic drugs. However, we did not collect these data in this study, which should be remedied in future studies. Fourth, previous studies found that there was sex difference in obesity in patients with schizophrenia^59^. However, in this study, we included much fewer female patients than male patients, which may have affected the results due to an imbalance in the sex ratio. Finally, our study did not have a healthy control group. Therefore, the results in this study should be considered preliminary.

## Conclusions

In summary, we demonstrated that high BMI was associated with lower Hcy levels, higher total cholesterol levels, and more severe psychopathological symptoms in patients with chronic schizophrenia. Moreover, Hcy was negatively associated with high BMI in these schizophrenia patients. Also, our results showed that high BMI patients had higher education levels but was not correlated with clozapine/olanzapine use. Future study in a large sample size with balanced male and female patients using a prospective and longitudinal design will be needed to explore the relationship between Hcy and obesity in first-episode and drug naïve patients with schizophrenia.

### Compliance with ethical standards

All procedures were based on the Declaration of Helsinki issued by the National Institutes of Health. The research scheme has been approved by the Ethics Committee of the Affiliated Brain Hospital of Guangzhou Medical University before the study began. Written informed consents were obtained from each subject before participating in this study.
